# Multi-contrast attenuation map synthesis for PET/MR scanners: assessment on FDG and Florbetapir PET tracers

**DOI:** 10.1007/s00259-015-3082-x

**Published:** 2015-06-24

**Authors:** Ninon Burgos, M. Jorge Cardoso, Kris Thielemans, Marc Modat, John Dickson, Jonathan M. Schott, David Atkinson, Simon R. Arridge, Brian F. Hutton, Sébastien Ourselin

**Affiliations:** 1 Translational Imaging Group, Centre for Medical Image Computing, University College London, NW1 2HE London, UK; 2 Dementia Research Centre, Institute of Neurology, University College London, WC1N 3AR London, UK; 3 Institute of Nuclear Medicine, University College London, NW1 2BU London, UK; 4 Centre for Medical Imaging, University College London, WC1E 6JF London, UK; 5 Centre for Medical Image Computing, University College London, WC1E 6BT London, UK; 6 Centre for Medical Radiation Physics, University of Wollongong, NSW 2522 Wollongong, Australia

**Keywords:** Image synthesis, Attenuation correction, PET/MR

## Abstract

Positron Emission Tomography/Magnetic Resonance Imaging (PET/MR) scanners are expected to offer a new range of clinical applications. Attenuation correction is an essential requirement for quantification of PET data but MRI images do not directly provide a patient-specific attenuation map.

*Methods* We further validate and extend a Computed Tomography (CT) and attenuation map (*μ*-map) synthesis method based on pre-acquired MRI-CT image pairs. The validation consists of comparing the CT images synthesised with the proposed method to the original CT images. PET images were acquired using two different tracers (^18^F-FDG and ^18^F-florbetapir). They were then reconstructed and corrected for attenuation using the synthetic *μ*-maps and compared to the reference PET images corrected with the CT-based *μ*-maps. During the validation, we observed that the CT synthesis was inaccurate in areas such as the neck and the cerebellum, and propose a refinement to mitigate these problems, as well as an extension of the method to multi-contrast MRI data.

*Results* With the improvements proposed, a significant enhancement in CT synthesis, which results in a reduced absolute error and a decrease in the bias when reconstructing PET images, was observed. For both tracers, on average, the absolute difference between the reference PET images and the PET images corrected with the proposed method was less than 2%, with a bias inferior to 1%.

*Conclusion* With the proposed method, attenuation information can be accurately derived from MRI images by synthesising CT using routine anatomical sequences. MRI sequences, or combination of sequences, can be used to synthesise CT images, as long as they provide sufficient anatomical information.

****Electronic Supplementary Material**:**

The online version of this article (doi:10.1007/s00259-015-3082-x) contains supplementary material, which is available to authorized users.

## Introduction

Recent clinical studies have shown the advantages of Positron Emission Tomography/Magnetic Resonance (PET/MR) imaging in neuro-oncology [1], epilepsy [2] and neurodegenerative diseases such as Alzheimer’s disease (AD) [3]. Correcting for photon attenuation is essential to accurately quantify the radionuclide uptake, especially in neuroimaging where the skull attenuation coefficients are high. In the absence of a transmission source, Computed Tomography (CT) image or time-of-flight PET, the attenuation information can only be derived from MRI images. However, MRI image intensities do not reflect the electron density, which prevents a direct estimation of the attenuation coefficients. With MRI images, a specific challenge is to differentiate between bone and air as they often both have low intensity. A lack of accuracy in the bone delineation has been shown to lead to a strong spatial bias of the PET activity [4].

MRI-based attenuation correction methods can be classified in two main categories: segmentation and registration-based approaches [5]. The segmentation-based strategy consists of assigning uniform linear attenuation coefficients to tissue classes obtained by segmenting a T1-weighted MRI image [6], or images derived from Dixon [7] and/or Ultrashort-Echo-Time (UTE) sequences [8–12]. In the brain, the use of Dixon sequences is limited to water-fat separation while UTE sequences allow cortical bone segmentation. This class of methods can produce inaccuracies in areas such as the sinuses, where differentiation between bone and air is required, and the resulting attenuation maps do not reflect the range of attenuation values present in the body.

In registration-based methods, an attenuation map (*μ*-map) template, derived from pre-acquired CT or transmission images, is deformed to match the patient’s anatomy. In the method from [13], the CT template is directly registered to the patient MRI image while in other methods [14–17], an MRI image is associated with the *μ*-map template and the mapping is performed between the template and patient MRI images. Used on its own, image registration proves to be insufficient to generate accurate *μ*-maps but has shown promising results when associated with a Gaussian process [15] or a voxel-wise weighting scheme [16, 17].

Combining segmentation and registration, Izquierdo-Garcia et al. [18] propose to generate attenuation maps using the Statistical Parametric Mapping (SPM) software. The patient’s MRI image is first segmented into 6 tissue classes and then non-rigidly registered to a segmented MRI template. The CT template, aligned with the MRI template, is finally mapped into the patient’s space by applying the inverse transformation.

In this paper, we validate a CT and attenuation map synthesis algorithm based on a multi-atlas information propagation scheme developed by Burgos et al. [16, 17] and compare it to a state-of-the-art method [18] based on a package widely used in the neuro-imaging community (SPM). In order to mitigate some limitations that we observed, we also propose a better estimation of the local image similarity and an extension of the method to multi-contrast MRI data. The method was validated with 22 subjects with various dementia syndromes, who had MR imaging and two PET/CT scans with different tracers: ^18^F-FDG and ^18^F-florbetapir. The validation was done jointly using two PET tracers to confirm the independence of the method to the radiopharmaceutical administered. We assessed the accuracy of the CT synthesis by comparing to the original CT images. The PET reconstruction accuracy was assessed, for both tracers, by comparing the reference PET images, corrected for attenuation using the reference CT-based *μ*-map, to the PET images corrected using the proposed method. We analysed results both across the full brain but also in regions of particular interest, e.g. when studying dementia.

## Materials and methods

### PET/CT and MRI data acquisition

Twenty-two sets of ^18^F-FDG PET, ^18^F-florbetapir PET, CT and MRI brain images were used in order to validate our attenuation correction method. The 22 individuals (5 with posterior cortical atrophy, 5 with semantic dementia, 4 with progressive nonfluent aphasia, 3 with logopenic progressive aphasia and 5 healthy controls) each attended for three imaging sessions on three consecutive days. At the first visit, MR imaging was acquired on a 3T Siemens Magnetom Trio scanner (Siemens Healthcare, Erlangen, Germany) and includes a T1-weighted magnetisation-prepared rapid gradient-echo (3.0 T; acquisition time 9 min 23 s; TE/TR/TI, 2.9 ms/2200 ms/900 ms; flip angle 10°; voxel size 1.1 × 1.1 × 1.1 mm^3^) and a T2-weighted (3.0 T; acquisition time 4 min 43 s; TE/TR, 401 ms/3200 ms; flip angle 120°; voxel size 1.1 × 1.1 × 1.1 mm^3^) volumetric scans. For the second and third visits, imaging was performed on a GE Discovery ST PET/CT scanner (GE Healthcare systems, Waukesha, WI), providing CT (voxel size 0.59 × 0.59 × 2.5 mm^3^, 120 kVp, 300 mA) and PET (voxel size 1.95 × 1.95 × 3.27 mm^3^) images. For visit two, images were acquired for 10 minutes, 50 minutes after injection of 200 MBq ^18^F-florbetapir; for visit three, images were acquired for 20 minutes, 30 minutes after injection of 185 MBq ^18^F-FDG. The local ethics committee approved the study and all subjects gave written, informed consent.

Because of the separate PET and MRI acquisitions, sequences used for MR-based attenuation correction, such as Dixon or UTE sequences, were not acquired. We refer the reader to [17] for a comparison between the CT synthesis and UTE-based methods.

### Validation on ^18^F-FDG and ^18^F-florbetapir PET tracers

The aim of this paper is to validate the MR-based attenuation correction method proposed by [17] for ^18^F-FDG and ^18^F-florbetapir PET tracers. To do so, pseudo CT (pCT) images, synthesised from MRI images as explained in section “CT synthesis”, were used during the reconstruction of PET images to correct for attenuation.

A common practice in the neuroimaging community is to normalise PET images using a reference region [19, 20]. For the FDG PETs, the mean uptake value in the pons [19] was used to normalise the PET images of each subject, thus allowing for a comparable range of values. For the florbetapir PETs, the mean value in the cerebellar grey matter [20] was used. These reference regions were extracted from parcellated T1-weighted MRI images. The parcellations were obtained from a multi-label fusion algorithm, as implemented in NiftySeg [21].

Once the PET images were normalised, the analysis consisted in providing quantitative regional assessment of the mean absolute error and the mean voxel bias between the PET images corrected with the method proposed and the PET images corrected with the attenuations maps derived from the reference CT images. The full brain region, posterior cingulate gyrus, angular gyrus, superior frontal gyrus, fusiform gyrus and anterior cingulate gyrus, regions relevant to dementia pathologies, were obtained from the parcellated T1-weighted MRI images and propagated to the PET images.

### CT synthesis

The CT synthesis method developed in [17] relies on a pre-acquired set of aligned MRI/CT image pairs from multiple subjects forming an MRI-CT database. To generate the CT from a target MRI image, each MRI image from the database are deformed to the target MRI image using affine followed by non-rigid registration [22]. The CT images in the database are then mapped using the same transformation to the target MRI image. A local image similarity measure [23] between the target MRI and the set of registered MRIs from the database is used as a surrogate of the underlying morphological similarity, under the assumption that, if two MRIs are similar at a certain spatial location, the two CTs will also be similar at this location. To generate the pseudo CT, the set of registered CTs is fused using a voxel-wise weighting scheme [21, 24]. Finally, the CT values, expressed in HU, are converted to linear attenuation coefficients, in cm^−1^, using a piecewise linear transformation [25]. The resulting attenuation maps are smoothed using a Gaussian filter with a kernel standard deviation of 2 voxels (1.172 × 1.172 × 2.5 mm^3^) to approximate the PET’s point spread function (PSF), and resampled to the PET’s discretisation grid.

While providing an accurate *μ*-map synthesis, we found through experimentations (see section “Results”) that the method described in [17] had a few limitations related to the field-of-view (FOV) and the lack of complementary information, resulting in localised reconstruction inaccuracies close to critical areas used for standard uptake value ratio (SUVR) normalisation. Thus, rather than only validating the method in [17] for two tracers, we developed novel methodologies to address these limitations.

#### Convolution-based ROI-LNCC

The FOV of the MRI images usually contains the head and neck of the subject, while in the CT FOV only the head is visible, which can lead to mismatching information when images from the two modalities are aligned. A similar mismatch can occur with inter-subject mapping. If the mismatch between FOVs is not taken into account, the inter-subject mapping and resampling processes introduce areas where no information is available, which can lead to severe underestimation of the *μ*-map. Those areas have to be accounted for when the similarity measure is computed and during the intensity fusion process. We extend the convolution-based local normalised correlation coefficient (LNCC) method by Cachier et al.[23] to irregular regions-of-interest (ROI) (see Appendix A). Thanks to this process, LNCC values are only valid within the bounds of the FOV. The ROI-LNCC at each voxel is then ranked across all atlas images and the ranks are converted to weights by applying an exponential decay function. These weights are used in a spatially varying weighted averaging to reconstruct the target CT [17].

#### Exploiting MRI multiple contrasts

The algorithm developed by [17] relies on the ability to accurately map T1 brain images from different subjects, a process that can be challenging in low-contrast areas such as the sinuses and the bone/dura/cerebrospinal fluid (CSF) boundary. As T1-weighted and T2-weighted MRI sequences provide complementary information to describe the underlying subject’s anatomy, we propose to combine information from both sequences at the registration and image similarity stages.

To do so, T1 and T2 images are affinely aligned to form a T1-T2 pair. The inter-subject coordinate mapping is obtained using a symmetric global registration followed by a cubic B-spline parametrised non-rigid registration, using multivariate normalised mutual information, as implemented in NiftyReg [22]. All the CTs in the database are then mapped to the target subject using the transformation that maps the subject’s corresponding MRI pair in the database to the target MRI. The multivariate ROI-LNCC used for the local atlas ranking procedure is here defined as the sum of the ROI-LNCC of each channel (Appendix 1B).

#### SPM-based approach

For comparison purposes, we implemented the approach presented by Izquierdo-Garcia et al. [18]. The initial step consists of creating an MRI and a CT template. To do so, the T1-weighted MRI images from the MRI-CT database are first segmented into 6 tissue classes (grey matter, white matter, CSF, bone, soft tissue, and air) using SPM12 [26]. The 22 segmented images are then non-rigidly co-registered using Dartel [27] to form the MRI template. The same transformations are applied to the CT images from the MRI-CT database, and the CT template is created by averaging the 22 co-registered CT images. To generate a pseudo CT, the patient’s MRI image is segmented into 6 tissue classes and non-rigidly registered to the MRI template. The associated CT template is finally mapped into the patient’s space by applying the inverse transformation.

### PET reconstruction

We used the PET images provided by the PET/CT scanner as input for a simulation technique. To evaluate the effect of different *μ*-maps on the PET images, we followed a projection/reconstruction technique described in [17]. The original PET image and the reference CT-based *μ*-map were projected to obtain simulated sinograms. The scatter sinogram was estimated from the emission data and the attenuation maps using a single scatter simulation algorithm [28]. Projection data were rescaled to account for attenuation and the estimated scatter sinogram was added to produce a non-corrected sinogram, similar to the data acquired by the PET/CT scanner. The non-corrected PET sinogram was then reconstructed with both scatter estimation and attenuation correction based on the reference CT or pseudo CT *μ*-maps. The PET image reconstructed using the reference CT-based *μ*-map was considered as the reference PET. An Ordered Subsets Expectation Maximisation (OSEM) algorithm with 3 iterations of 21 subsets was used. Effects of PSF and randoms were not included and post-reconstruction smoothing was not applied. The simulation and reconstruction were performed using STIR [29].

### Algorithmic comparison

The performance of the proposed synthesis algorithm was compared with ground truth data for 22 subjects following a leave-one-out cross-validation scheme. For each subject, and both PET tracers, 5 pseudo CTs were synthesised using a database of 21 subjects following the method from [17] and the improvements proposed. An additional pseudo CT was synthesised using the SPM-based approach [18]:

- pCT_SPM_ using the method described in [18] given the T1 image;
- pCT_T1_ using the method described in [17] given the T1 image;
- pCT_T2_ using the method described in [17] given the T2 image;
- npCT_T1_ using the ROI-LNCC on the T1 image;
- npCT_T2_ using the ROI-LNCC on the T2 image;
- npCT_T1,T2_ using the MV-ROI-LNCC on the T1-T2 pair of images.

In order to preserve the alignment of the CT and PET images, the two PET/CT acquisitions were considered independently, meaning that we created two MR-CT databases: one with the CT images from the ^18^F-FDG PET/CT scan and the MR images; and another one with the CT images from the ^18^F-florbetapir PET/CT scan and the MR images.

The quantitative validation consisted of two steps:

1. The pseudo CTs were compared to the subject’s original CT image, validating the accuracy of the CT synthesis.
2. For both FDG and florbetapir tracers, the simulated PET data were reconstructed using the different pCT *μ*-maps, and compared with the reference PET reconstructed using the CT-based *μ*-map, validating the accuracy of the PET attenuation correction.

Statistical significance was assessed using the paired one-tailed Wilcoxon signed-rank test, with a 5% significance level.

#### Pseudo CT accuracy

For every subject, the mean absolute error and the mean error, defined respectively as

1 $$\begin{array}{@{}rcl@{}} \text{MAE}&=&\frac{1}{N_{V}} {\sum\limits}_{v\in V}|I_{v}-R_{v}| \;\;\;\text{and} \\ \text{ME}&=&\frac{1}{N_{V}} {\sum\limits}_{v\in V}{\left( I_{v}-R_{v}\right)} , \end{array} $$

were calculated between the reference CT (*R* = *C* *T*) and each of the pseudo CTs (*I* = *p* *C* *T*), in a region of interest *V* comprising *N*_*V*_ voxels. This region of interest is limited to a mask defined by segmenting the head from the background using the original CT. The MAE provides information about reconstruction error and deviations from the expected values while the ME gives information about an inherent bias in the methodology.

To localise the error and bias introduced by each approach, the original CTs and pseudo CTs from the 22 subjects were mapped to a common space via a groupwise registration [30]. Difference maps were then computed between the original CT and the pseudo CTs and averaged across all the subjects. Results are presented using a mean intensity projection: the mean value along a projection line is assigned to the pixel represented on the projected image.

#### PET accuracy

We first computed the relative MAE and ME in the reference regions, defined respectively as

2 $$\begin{array}{@{}rcl@{}} \text{rMAE}&=&100*\frac{{\sum\limits}_{v\in V}{|I_{v}-R_{v}|}}{{\sum}_{v\in V}{R_{v}}}\;\;\;\; \text{and} \\ \text{rME}&=&100*\frac{{\sum\limits}_{v\in V}{\left( I_{v}-R_{v}\right)}}{{\sum}_{v\in V}{R_{v}}} \;\;\;, \end{array} $$

between the reference PET (*R* = *P* *E* *T*_*CT*,*ref*_) and each of the PETs corrected with the synthetic *μ*-maps (*I* = *P* *E* *T*_*pCT*,*ref*_). This analysis aims at characterising the presence of error and bias in the reference regions.

In order to provide a quantitative regional assessment of the error and bias after normalisation by the reference region, we computed the relative MAE and ME between the normalised reference PET (*R* = *P* *E* *T*_*CT*_/*m* *e* *a* *n*_*ref*_) and each of the PETs corrected with the synthetic *μ*-maps (*I* = *P* *E* *T*_*pCT*_/*m* *e* *a* *n*_*ref*_). To assess the performance of the proposed method in areas relevant to dementia pathologies, we analysed results in the full brain region, but also in the posterior cingulate gyrus, angular gyrus, superior frontal gyrus, fusiform gyrus and anterior cingulate gyrus.

Finally, the PET images from the 22 subjects were mapped to a common space via a CT-based groupwise registration [30]. Difference maps were then computed between the reference PET and each of the PETs corrected with the pCT *μ*-maps, and their average and standard deviation, across all the subjects, displayed using a mean intensity projection.

## Results

### Pseudo CT accuracy

**Fig. 1:**
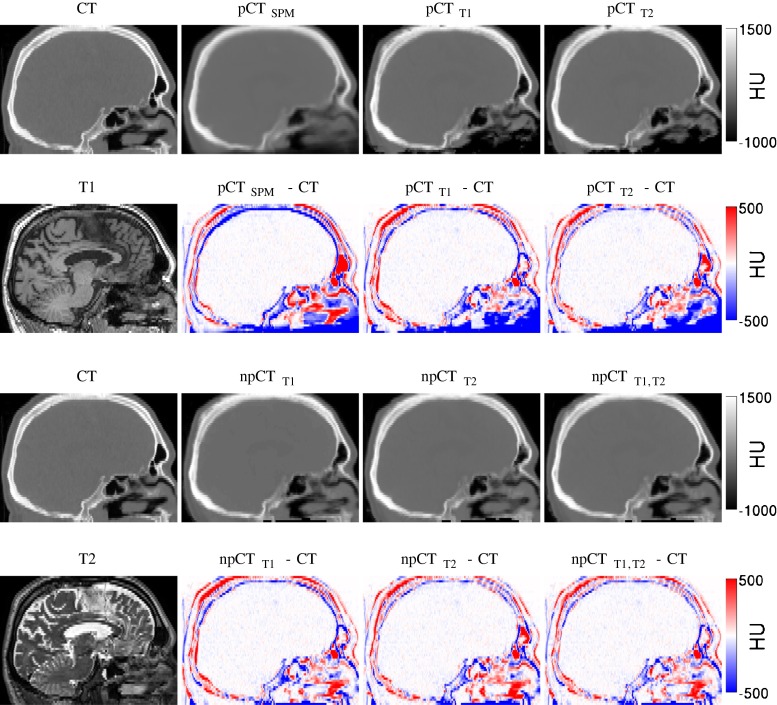
For a representative subject, the acquired CT, T1 and T2 images (left), and the pseudo CTs with the associated difference images

The average, standard deviation (SD), minimum and maximum values of the MAEs and MEs computed in the full head are presented in Table 1. We note that the MAE is smaller with the proposed method than with the SPM-based approach. Using the ROI-LNCC instead of the classical LNCC [17] significantly (*p* < 10^−4^) decreases the MAE with further reduction in MAE (*p* < 10^−4^) when exploiting the multi-contrast approach. By combining these two steps, the CT synthesis error is decreased, on average, by 30%. The ME results presented in Table 1 show that using the ROI-LNCC instead of the classical LNCC also reduces the bias. An example of original CT and pseudo CTs is presented in Fig. 1 for the FDG cohort.

**Table 1:** Mean, standard deviation (SD), minimum and maximum of the MAE and ME computed for the full head between the reference CT and the pseudo CTs for the ^18^F-FDG and ^18^F-florbetapir PET/CT scans

	FDG cohort								Florbetapir cohort							
	MAE (HU)				ME (HU)				MAE (HU)				ME (HU)			
	Mean	SD	Min	Max	Mean	SD	Min	Max	Mean	SD	Min	Max	Mean	SD	Min	Max
pCT_SPM_	130	33	95	246	−24	27	−61	49	129	32	90	244	−23	25	−62	49
pCT_T1_	122	21	90	158	−39	22	−77	10	118	22	82	161	−33	19	−63	0
pCT_T2_	119	21	87	162	−33	25	−69	17	116	20	84	158	−29	22	−67	5
npCT_T1_	89	11	73	122	3	16	−24	44	89	11	70	126	4	14	−21	30
npCT_T2_	88	9	75	107	7	17	−18	53	88	8	76	102	7	14	−16	37
npCT_T1,T2_	82	8	69	101	4	15	−19	47	82	7	70	97	4	13	−14	32

Note that the difference observed between the FDG and florbetapir cohorts (Table 1) is small and is only due to differences in the affine alignment between the CT and MRI images used to form the two databases. Using a single database would have been sufficient but we chose to process the two PET/CT acquisitions separately to maintain the alignment of the CT and PET images, and thus not favour an acquisition while analysing the PET results.

**Fig. 2:**
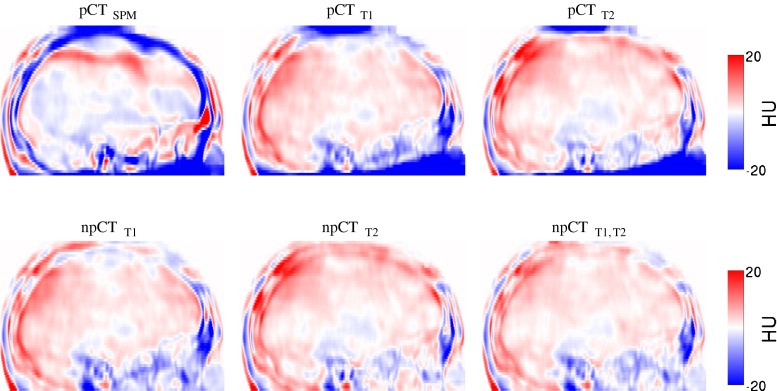
Mean intensity projection of the difference, averaged over 22 subjects, between the reference CT and the pseudo CTs

The difference maps computed between the original CT and the pseudo CTs, and averaged across all the subjects, are presented in Figure 2 for the ^18^F-FDG PET/CT scan. Large errors at the top and bottom of the head, when the LNCC of [17] is used, disappear when the FOV are properly handled as described in section “Convolution-based ROI-LNCC”. Regardless of the method applied, the largest errors are now mostly located in the sinus area and at the bone/dura/CSF boundary. Similar results were obtained for the ^18^F-florbetapir PET/CT scan.

**Table 2:** Average ± SD of the relative MAE and ME between the ^18^F-FDG and ^18^F-florbetapir PETs corrected with the CT-based *μ*-maps and the PETs corrected with the synthetic *μ*-maps in 7 ROIs: the reference region used for normalisation, the brain region, angular gyrus (AG), superior frontal gyrus (SFG), posterior cingulate gyrus (PCG), fusiform gyrus (FG) and anterior cingulate gyrus (ACG)

			pCT_SPM_	pCT_T1_	pCT_T2_	npCT_T1_	npCT_T2_	npCT_T1,T2_
FDG	Ref	rMAE (%)	2.38 ± 1.12	1.88 ± 0.71	1.81 ± 0.75	1.79 ± 0.64	1.72 ± 0.62	1.64 ± 0.60
		rME (%)	0.65 ± 2.04	−0.40 ± 1.26	−0.14 ± 1.16	−0.12 ± 1.37	0.09 ± 1.23	-0.15 ± 1.21
	Brain	rMAE (%)	2.76 ± 1.13	2.25 ± 0.75	2.09 ± 0.72	1.96 ± 0.56	1.85 ± 0.64	1.71 ± 0.62
		rME (%)	−0.51 ± 1.61	0.46 ± 1.06	0.41 ± 0.58	0.58 ± 0.97	0.54 ± 1.08	0.56 ± 0.98
	AG	rMAE (%)	2.93 ± 1.60	2.99 ± 2.36	2.48 ± 1.54	2.79 ± 1.91	2.32 ± 1.45	2.10 ± 1.41
		rME(%)	−0.84 ± 2.95	1.83 ± 3.09	1.37 ± 2.46	1.58 ± 2.69	1.17 ± 2.33	0.98 ± 2.17
	SFG	rMAE (%)	3.63 ± 3.04	2.16 ± 1.26	1.72 ± 1.07	2.01 ± 1.17	1.66 ± 1.02	1.53 ± 1.01
		rME (%)	−0.37 ± 3.49	0.61 ± 2.10	0.90 ± 1.65	0.38 ± 1.95	0.69 ± 1.65	0.57 ± 1.58
	PCG	rMAE (%)	1.56 ± 1.09	1.21 ± 0.85	1.14 ± 0.79	1.11 ± 0.73	1.10 ± 0.74	1.06 ± 0.74
		rME (%)	−0.62 ± 1.79	0.74 ± 1.24	0.59 ± 1.23	0.50 ± 1.19	0.41 ± 1.24	0.44 ± 1.20
	FG	rMAE (%)	2.23 ± 1.09	1.87 ± 0.59	1.91 ± 0.63	1.83 ± 0.56	1.84 ± 0.63	1.68 ± 0.57
		rME (%)	−0.59 ± 1.85	0.78 ± 1.17	0.40 ± 1.31	0.71 ± 1.17	0.38 ± 1.27	0.55 ± 1.12
	ACG	rMAE (%)	1.57 ± 1.01	1.23 ± 0.70	1.08 ± 0.75	1.14 ± 0.60	1.09 ± 0.69	1.05 ± 0.66
		rME (%)	−0.49 ± 1.76	0.64 ± 1.19	0.49 ± 1.16	0.36 ± 1.18	0.27 ± 1.21	0.39 ± 1.14
Florbetapir	Ref	rMAE (%)	2.73 ± 0.62	2.74 ± 0.95	2.77 ± 1.09	2.02 ± 0.59	2.11 ± 0.74	1.93 ± 0.62
		rME (%)	−0.74 ± 1.51	−1.05 ± 1.24	−0.95 ± 1.48	0.42 ± 1.21	0.50 ± 1.36	0.39 ± 1.20
	Brain	rMAE (%)	2.76 ± 1.13	2.56 ± 1.17	2.38 ± 1.14	1.87 ± 0.39	1.74 ± 0.48	1.60 ± 0.41
		rME (%)	0.94 ± 1.45	1.27 ± 1.49	1.24 ± 1.40	0.04 ± 0.92	0.02 ± 0.99	−0.02 ± 0.87
	AG	rMAE (%)	2.82 ± 1.54	3.32 ± 2.43	2.48 ± 1.64	2.67 ± 1.43	1.90 ± 1.05	1.82 ± 0.94
		rME (%)	0.76 ± 2.85	2.16 ± 3.25	1.71 ± 2.28	0.68 ± 2.53	0.27 ± 1.91	0.06 ± 1.77
	SFG	rMAE (%)	3.62 ± 4.13	2.36 ± 1.36	2.12 ± 1.32	1.86 ± 0.78	1.50 ± 0.85	1.38 ± 0.72
		rME (%)	1.43 ± 4.45	1.18 ± 2.22	1.42 ± 1.93	−0.33 ± 1.66	−0.07 ± 1.54	−0.28 ± 1.34
	PCG	rMAE (%)	1.46 ± 0.93	1.66 ± 1.21	1.62 ± 1.22	1.07 ± 0.53	1.09 ± 0.69	1.05 ± 0.59
		rME (%)	0.65 ± 1.59	1.34 ± 1.55	1.27 ± 1.57	−0.11 ± 1.16	−0.15 ± 1.24	−0.17 ± 1.16
	FG	rMAE (%)	2.20 ± 0.90	2.24 ± 1.15	2.07 ± 1.05	1.57 ± 0.36	1.58 ± 0.41	1.49 ± 0.36
		rME (%)	0.72 ± 1.50	1.64 ± 1.50	1.33 ± 1.41	0.25 ± 0.92	−0.02 ± 0.89	0.11 ± 0.85
	ACG	rMAE (%)	1.61 ± 1.04	1.59 ± 1.15	1.62 ± 1.21	1.11 ± 0.58	1.20 ± 0.82	1.04 ± 0.68
		rME (%)	0.89 ± 1.63	1.22 ± 1.47	1.21 ± 1.56	−0.27 ± 1.16	−0.26 ± 1.37	−0.22 ± 1.17

### PET accuracy

**Fig. 3:**
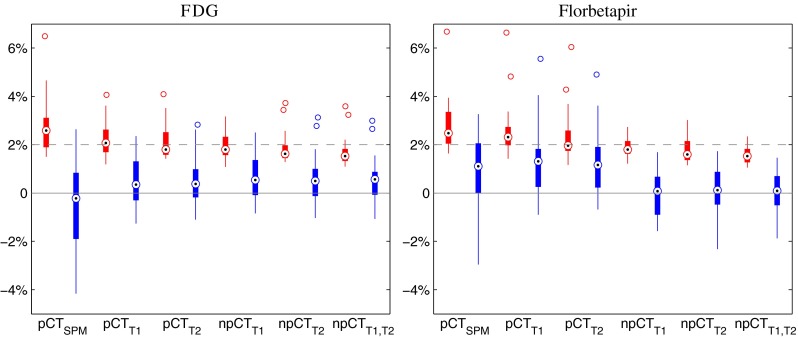
Boxplots displaying the median, lower and upper quartiles, minimum, maximum and outliers of the rMAE (red) and rME (blue) calculated between the reference PETs and the PETs corrected with the synthetic *μ*-maps, in the brain region for both tracers

The average and SD of the rMAEs and rMEs are presented in Table 2. Results for the brain region are also displayed with box plots in Fig. 3. Box plots displaying results in the other ROIs are available in the supplemental data (Figs. S1 and S2).

Errors in the reference regions are lower for the FDG PET than for the florbetapir PET as the regions surrounding the pons are better synthesised than the cerebellum, which is closer to the border of the FOV. In both cases, with the improvements proposed, the error is less than 2%. Note that, when using the method from [17], the larger errors in the cerebellum will lead to normalisation-derived inaccuracies in the full brain.

**Fig. 4:**
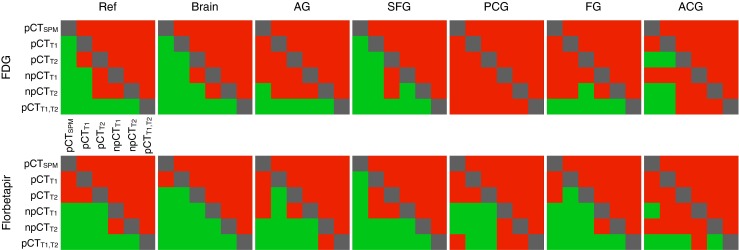
Results of the one-tailed Wilcoxon signed-rank test at the 5% significance level for the rMAE computed between the reference PET and each of the PETs corrected with the pCT *μ*-maps in the reference regions, the full brain and 5 ROIs. The colour green indicates a significant decrease in rMAE for the row method when compared to the column method, while the red indicates that the difference in rMAE is not significant. Note that, for most ROIs, npCT_T1,T2_ is significantly better

For both tracers, in the brain region, the rMAE is smaller with the proposed method compared to the SPM-based approach. When the ROI-LNCC similarity measure is computed instead of the standard LNCC [17], the rMAE significantly decreases. Exploiting MRI multiple contrasts further reduces the rMAE. By combining the two improvements, the PET reconstruction error in the brain is on average decreased, by 25% for the FDG tracer and 40% for the florbetapir tracer, and remains below 2% (Fig. 3). In ROIs close to the skull (superior frontal gyrus, angular gyrus and fusiform gyrus), the rMAE does not exceed 3%, while in deeper structures (posterior cingulate gyrus, and anterior cingulate gyrus), with the new improvements, the rMAE is below 1.2%. A summary of the significance tests between the different methods is presented in Fig. 4.

In order to analyse the presence of bias in the PET images, we computed the rME between the reference PETs and the PETs corrected with the synthetic *μ*-maps. In the full brain region, the rME results indicate a low bias: the rME is on average below 0.6% for the FDG tracer, and below 0.1% for the florbetapir tracer, with a standard deviation of 1% for both tracers. For the FDG tracer, the region presenting the greatest bias is the angular gyrus with an ME of 0.98 ± 2.17%. For the florbetapir tracer, the superior frontal gyrus presents the greatest bias, with an ME of -0.28 ± 1.34%.

**Fig. 5:**
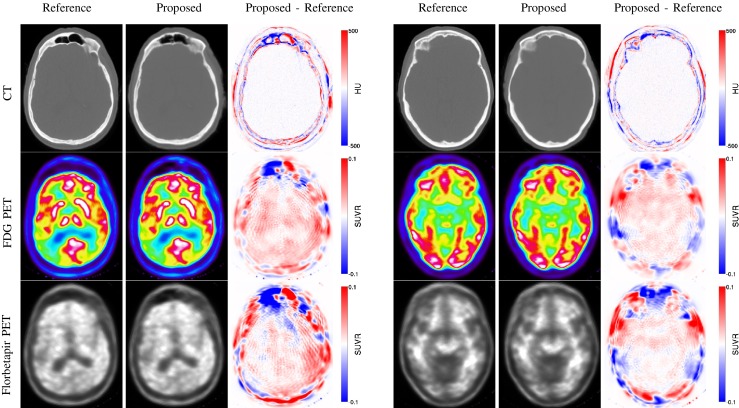
Example of CT (top row), ^18^F-FDG PET (middle row) and ^18^F-florbetapir PET (bottom row) images obtained from the reference and pseudo CTs, and difference images, for an amyloid positive (left) and an amyloid negative (right) subjects

An example of PET images reconstructed with the CT-based *μ*-maps and synthetic *μ*-maps (npCT_T1,T2_) is presented in Fig. 5.

**Fig. 6:**
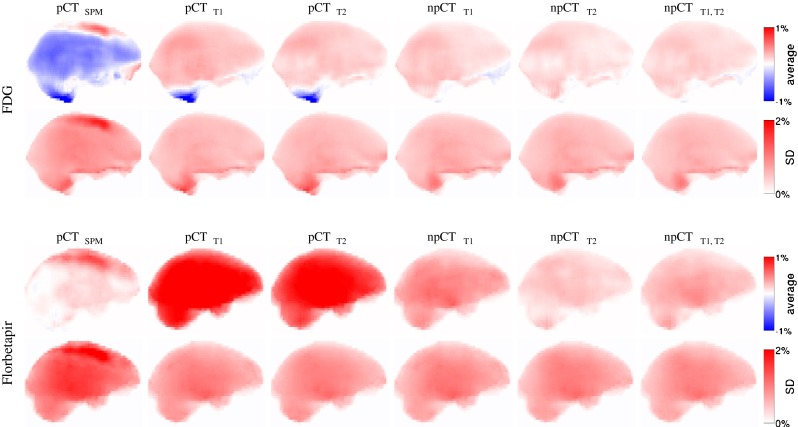
Mean intensity projection of the average and SD over 22 subjects of the difference maps. The difference maps were computed between the normalised ^18^F-FDG (top) and ^18^F-florbetapir (bottom) reference PETs and the PETs corrected with the synthetic *μ*-maps

The average and SD across all the subjects of the difference maps computed between the normalised reference PETs and PETs_pCT_ are presented in Fig. 6. Large errors are visible in the cerebellum when the LNCC of [17] is used (Fig 6, pCT_T1_ & pCT_T2_) but are reduced when the ROIs are properly handled as described in section “Convolution-based ROI-LNCC” (Fig 6, npCT_T1_ & npCT_T2_). Moreover, the error decreases further when the multi-contrast data are used (Fig 6, npCT_T1,T2_). Note that, as the cerebellar grey matter is used to normalise the ^18^F-florbetapir PET images, the presence of bias in this area leads to major activity misestimations in the full brain (Fig 6, Florbetapir, pCT_T1_ & pCT_T2_).

## Discussion

Correcting for attenuation is an essential requirement to perform an accurate quantitative analysis of PET data. In this paper, we jointly validated an MR-based attenuation correction method on two different tracers using 22 subjects with dementia. Each subject underwent two PET/CT scans, with ^18^F-FDG and ^18^F-florbetapir tracers, providing reference CT and PET images, and an MRI scan providing T1-weighted and T2-weighted images. The evaluation was conducted both in the full brain and in ROIs relevant for dementia (posterior cingulate gyrus, angular gyrus, superior frontal gyrus, fusiform gyrus and anterior cingulate gyrus).

While validating the method using the pseudo CTs from [17], we noticed problems in locations close to the edges of the template database field-of-view, such as the areas surrounding the cerebellum. This was problematic because the cerebellum was used as a SUVR normalising region. By introducing some methodological improvements, we were able to improve the CT synthesis accuracy. First, we proposed a new similarity measure for irregular regions-of-interest (ROI-LNCC) to increase the accuracy of the synthesis at the borders of the field-of-view. As a second step, we extended the method from [17] to multi-contrast MR data, allowing the introduction of complementary information. We showed that one can synthesise CT from T1 images, T2 images, but also combinations of MR contrasts. Combining complementary information describing the underlying subject’s anatomy, at both the registration and image similarity stages, reduces the ill-posedness of the problem. As a consequence, the CT synthesis error decreases, which leads to reduced errors when comparing PET images to the reference PETs. By combining these two improvements, the average MAE in the full brain for the florbetapir PET images decreased from 2.56% to 1.60%, with a reduction in MAE variance from 1.17% to 0.41% (Table 2).

When analysing the results of the CT synthesis, we note that the errors are mostly located in the sinus area and at the bone/dura/CSF boundaries (Fig. 2), regions with a low tissue discriminative power. We demonstrated that, for both tracers, in the brain region, the error when comparing PET images corrected with the pseudo CTs to the reference PET images corrected using the original CT is less than 2% (Table 2). While in ROIs close to the skull (superior frontal gyrus, angular gyrus and fusiform gyrus), the MAE can be up to 3%, in deeper structures (posterior cingulate gyrus, and anterior cingulate gyrus), the MAE is below 1.2%. The variance in SUVR explained by the attenuation correction error is likely to be smaller than the intrinsic PET noise variance [31].

We also compared our approach to a state-of-the-art, template-based, method using SPM. The pseudo CT is generated by non-rigidly registering the segmented patient’s T1 image to a segmented T1 template and by applying the inverse transformation to the associated CT template. Generating the MRI and CT templates through a co-registration and averaging process leads to smooth pseudo CT images. Moreover, the outcome of the process depends on a single registration, which might be inaccurate. As a result, for both PET tracers, the proposed method outperforms the SPM-based approach.

To the best of our knowledge, this is the first time that an MR-based attenuation correction method has been jointly validated with two different PET tracers. While the FDG PET images were only moderately affected by the inaccuracies at the border of the FOV of the original pseudo CTs [17], these inaccuracies had a major impact on the florbetapir PET images as they were normalised using the cerebellar grey matter. With the improvements proposed, CT images were more accurately synthesised at the borders of the FOV, which includes the cerebellum area, thus reducing the errors observed in the florbetapir PET images. Our validation showed that, for both tracers, the PET images were accurately corrected for attenuation.

The proposed method does not require the acquisition of PET analysis specific MRI sequences, such as Dixon or UTE, which means that the acquisition protocol can be entirely dedicated to clinically-relevant sequences. In this paper, we used T1 and T2 images as these sequences are usually acquired as part of many standard acquisition protocols, but the method could be extended to any combination of sequences providing enough structural information and structural contrast. The resolution of both the T1 and T2 images was high (1.1 mm isotropic), which might not always be the case in clinical practice. For example, if the resolution of the T2 image is lower than the resolution of the T1 image, its contribution to the synthesis process may be limited. The quality of the results obtained is likely to be between the quality reached when using two high-resolution images and the quality reached when only using one sequence.

The scope of applications of the proposed methodology exceeds the field of PET/MR. For example, *μ*-map synthesis methods can also be used to correct the PET images for attenuation when the radiation dose needs to be kept to a minimum, such as for paediatric subjects. Using an appropriate database, a pseudo CT could be synthesised from an MRI image acquired during a previous examination, then registered to the non corrected PET image and finally used to correct the PET data.

While we focused our work on brain applications using subjects who do not present unusual or highly abnormal skull anatomies, further experiments are required to validate the method on subjects with pathologies affecting regions critical for attenuation correction, such as the skull, and in other regions of the body. As long as the morphological variability is represented in the database and the registration between MRI pairs is sufficiently accurate, the technique could, in theory, be applied to other body parts. Furthermore, the inclusion of clinical information (patient’s gender, age, weight or ethnicity), as suggested by [32], could be used to improve the bone-density estimates.

## Conclusion

This paper presents a validation on ^18^F-FDG and ^18^F-florbetapir PET tracers of an improved CT and attenuation map synthesis method based on the work by Burgos et al. [17]. The difference between the PET images corrected for attenuation with the CT-based *μ*-maps and the PET images corrected using the synthetic *μ*-maps is, on average, less than 2% for both tracers. The proposed method can be beneficial for clinical practice as it does not require the acquisition of task-specific MRI sequences.

### Electronic supplementary material

Below is the link to the electronic supplementary material.

(156 KB)

(197 KB)

## Appendix A: Convolution-based LNCC applied to irregular regions-of-interest

Let the target subject’s MRI be denoted by *I* ^*M**R**I*^ and, for each of the *N* atlases in the database, let the mapped MRI images of atlas *n* be denoted by $J_{n}^{MRI}$. The ROI-LNCC between *I* ^*M**R**I*^ and $J_{n}^{MRI}$ at voxel *v* is then given by:

3 $$ \text{ROI-LNCC}_{n,v}=\frac{\langle I^{MRI},J_{n}^{MRI} \rangle_{v}} {\sigma(I^{MRI})_{v} \: \sigma(J_{n}^{MRI})_{v}}\;\; . $$

Let Ω be a density function equal to 1 where the fields of view overlap, and 0 otherwise. The means and standard deviations at voxel *v* are calculated using a Gaussian kernel $G_{\sigma _{G}}$ with standard deviation *σ*_*G*_ = 3 voxels through density normalised convolution:

$$\begin{array}{@{}rcl@{}} \overline{I}_{v}=\frac{\left[G_{\sigma_{G}}*I\right]_{v}}{\left[G_{\sigma_{G}}*{\Omega}\right]_{v}}\;\;\;\;\;\; \sigma(I)_{v}=\sqrt{\overline{I^{2}}_{v} -\overline{I}^{2}_{v} }\;\;\;\;\;\; \langle I,J \rangle_{v}=\overline{I \cdot J}_{v} -\overline{I}_{v}\cdot\overline{J}_{v} \;\;, \end{array} $$

where ∗ denotes the convolution operator and $G_{\sigma _{G}}*{\Omega }$ represents a density normalisation term that compensates for areas with missing information. As the values of LNCC are only valid within the bounds of the FOV, LNCC values outside the FOV are set to $-\infty $.

### Appendix B: Multivariate ROI-LNCC

The multivariate ROI-LNCC (MV-ROI-LNCC) used for the local atlas ranking procedure in the case of multiple MR constrats is here defined as

4 $$ \text{MV-ROI-LNCC}_{n}= \text{ROI-LNCC}\left( I^{T1},J_{n}^{T1} \right) + \text{ROI-LNCC}\left( I^{T2},J_{n}^{T2} \right) \;. $$
