# Supplementary material for: Multi-contrast attenuation map synthesis for PET/MR scanners: assessment on FDG and Florbetapir PET tracers
Source: Eur J Nucl Med Mol Imaging. 2015 Jun 24;42(9):1447–58. doi: 10.1007/s00259-015-3082-x (PMC4502321; doi:10.1007/s00259-015-3082-x)
Supplement: Supplementary file 1 — (156 KB) [file 259_2015_3082_MOESM1_ESM.pdf]

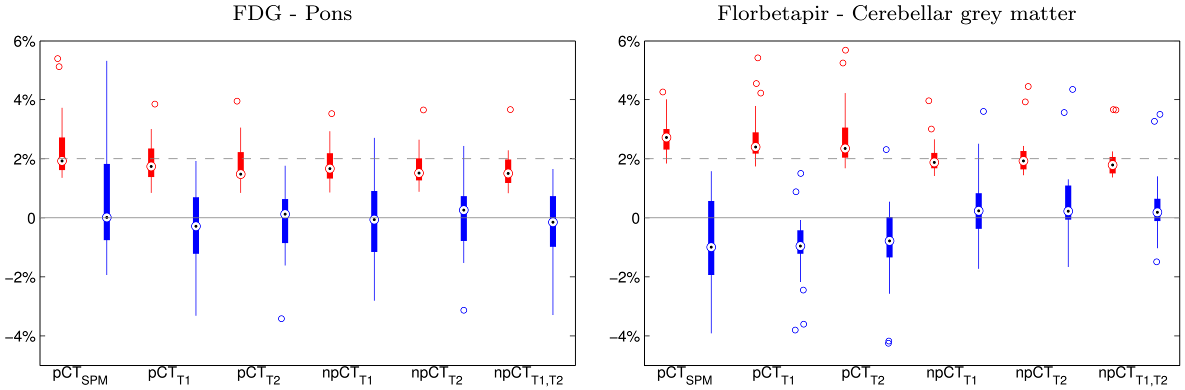

Fig. 1 Boxplots displaying the median, lower and upper quartiles, minimum, maximum and outliers of the rMAE (red) and rME (blue) calculated between the reference PETs and the PETs corrected with the synthetic μ-maps, for both tracers in the reference region used for normalisation.
