# Supplementary material for: Multi-contrast attenuation map synthesis for PET/MR scanners: assessment on FDG and Florbetapir PET tracers
Source: Eur J Nucl Med Mol Imaging. 2015 Jun 24;42(9):1447–58. doi: 10.1007/s00259-015-3082-x (PMC4502321; doi:10.1007/s00259-015-3082-x)
Supplement: Supplementary file 2 — (197 KB) [file 259_2015_3082_MOESM2_ESM.pdf]

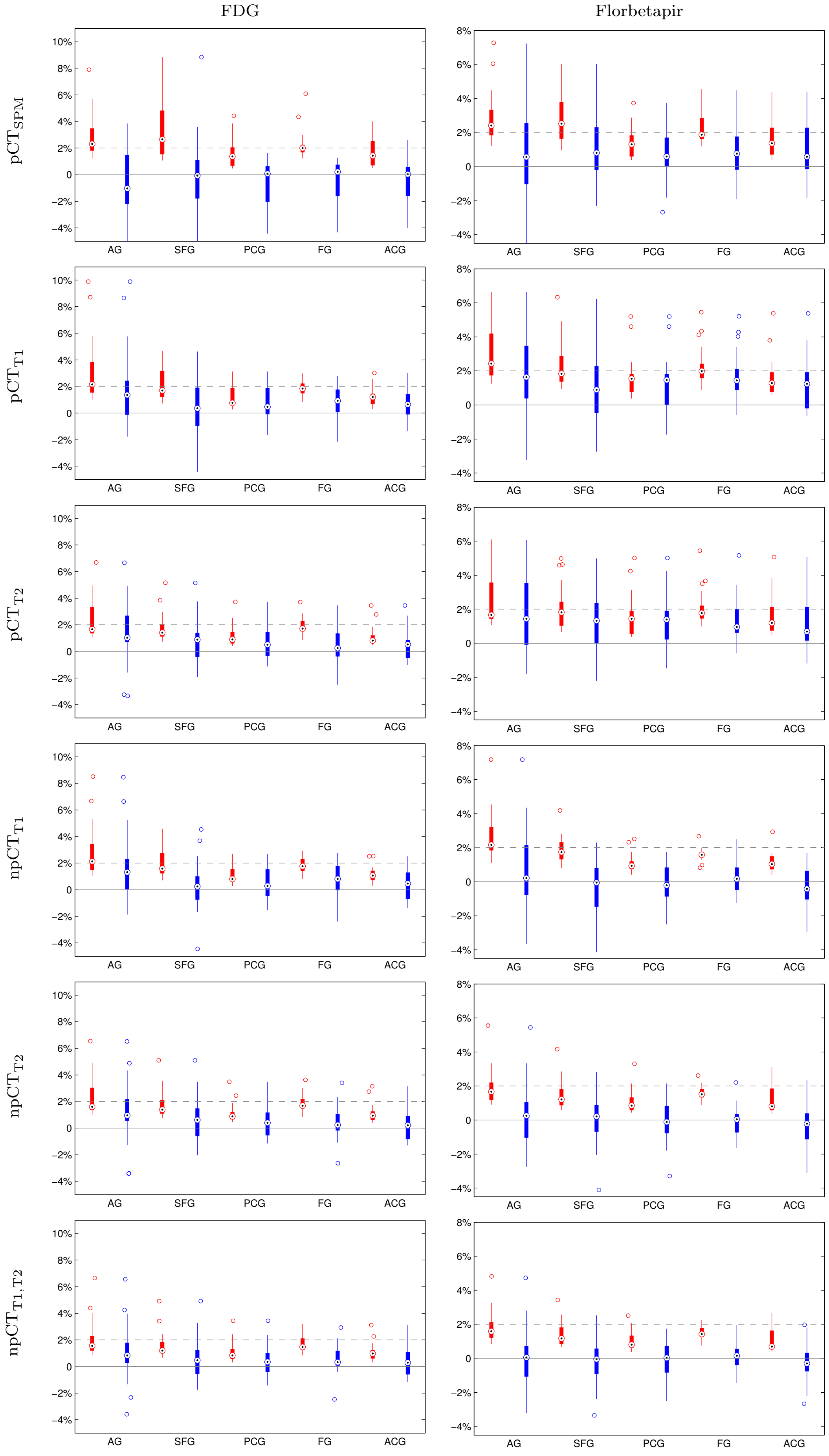

Fig. 2 Boxplots displaying the median, lower and upper quartiles, minimum, maximum and outliers of the rMAE (red) and rME (blue) calculated between the reference PETs and the PETs corrected with the synthetic μ-maps, for both tracers in 5 ROIS: angular gyrus (AG), superior frontal gyrus (SFG), posterior cingulate gyrus (PCG), fusiform gyrus (FG) and anterior cingulate gyrus (ACG).
